# The Kidney in the Shadow of Cirrhosis: A Critical Review of Renal Failure

**DOI:** 10.3390/biomedicines13112775

**Published:** 2025-11-13

**Authors:** Livia-Mirela Popa, Paula Anderco, Oana Stoia, Cristian Ichim, Corina Porr

**Affiliations:** Faculty of Medicine, Lucian Blaga University of Sibiu, 550169 Sibiu, Romania; liviamirelapopa@yahoo.com (L.-M.P.); cristian.ichim@ulbsibiu.ro (C.I.); corina.porr@ulbsibiu.ro (C.P.)

**Keywords:** hepatorenal syndrome, liver cirrhosis, acute kidney injury, biomarkers, acute tubular necrosis

## Abstract

Hepatorenal syndrome (HRS) is a high-mortality, potentially reversible form of kidney failure that arises from a tight hemodynamic–inflammatory coupling in cirrhosis. Contemporary redefinitions prioritize creatinine kinetics over static thresholds and recognize non-acute kidney injury (AKI) functional phenotypes, enabling earlier recognition but heightening the need for precise etiologic triage. This narrative synthesis integrates current concepts across pathophysiology, diagnosis and management. Portal hypertension, bacterial translocation and inflammatory mediators amplify splanchnic vasodilation and effective arterial underfilling. Compensatory neurohumoral activation precipitates renal vasoconstriction, intrarenal microcirculatory dysfunction and sodium–water retention. The pivotal diagnostic fork remains HRS–AKI versus acute tubular necrosis. A pragmatic, tiered strategy, structured volume assessment, filtration markers and a parsimonious tubular-injury panel offer actionable discrimination, whereas fractional excretion indices serve as adjuncts only. Initial therapy should be bundled and time-sensitive: remove nephrotoxins, treat infection and initiate albumin plus a vasoconstrictor. The transplant strategy should default to isolated liver transplantation unless end-stage renal disease is established. Future priorities include validated biomarker cut-offs, ultrasound-guided volume algorithms and pathway-based trials to reduce diagnostic delay and improve survival.

## 1. Introduction

Advanced chronic liver disease is often complicated by advanced kidney injury (AKI) and may be presented as prerenal AKI, hepatorenal syndrome (HRS) related AKI, intrinsic renal injury, most commonly acute tubular necrosis or post-renal obstruction (obstructive uropathies) [1]. Recent work has reshaped both the classification and the mechanistic understanding of AKI, revealing additional links between chronic liver disease and renal dysfunction [2]. Renal vasoconstriction and hemodynamic underfilling are not the sole drivers of HRS; mounting evidence implicates systemic inflammation, with increased pro-inflammatory cytokines, as a contributory pathway in the evolution of HRS [3,4].

In 1996, the International Club of Ascites specified that, in cirrhosis, AKI is present when serum creatinine increases by at least 50 percent from baseline to 1.5 mg/dL or higher, with low urine output and proteinuria below 500 mg/dL regarded as supportive features [5]. In 2007, HRS was divided into two types:

*Type 1:* Defined by a swift kidney decline over two weeks, either a doubling of serum creatinine to at least 2.5 mg/dL or a reduction of fifty percent or more in 24 h creatinine clearance to below 20 mL/min;

*Type 2:* For cases not meeting those thresholds, urinary sodium and oliguria are removed from the criteria [6].

Drawing on data showing that even a small jump in serum creatinine, about 0.3 mg/dL or a 50% rise from baseline, helps identify high-risk patients earlier, the International Club of Ascites updated the cirrhosis AKI definition and staging in 2015 [7,8,9]. The update also lets clinicians use a creatinine value from the previous three months when a 7-day baseline is not available [7,8,9]. Although oliguria was excluded, urine output correlated with adverse outcomes, prompting calls to broaden the 2015 framework [10,11].

Former “type 1 HRS” is now termed “HRS–AKI” and the minimum creatinine threshold was removed because higher creatinine at treatment initiation predicts lower reversal rates [12,13]. Hence, HRS–AKI can be diagnosed even below 2.5 mg/dL [8]. Functional renal impairment that fails to meet these thresholds is designated “HRS–non–AKI”. Within this category, cases are classified as “HRS–AKI” when the estimated glomerular filtration rate is under 60 mL/min/1.73 m^2^ for less than three months and as “HRS–chronic kidney disease” when it remains under 60 mL/min/1.73 m^2^ for more than three months [11].

A major clinical challenge in cirrhosis is distinguishing HRS-associated AKI from acute tubular necrosis. Although HRS is largely functional in nature, a kidney biopsy is seldom performed to confirm acute tubular necrosis [2]. Consequently, novel biomarkers are being explored to enable earlier and more accurate diagnosis and treatment initiation. Candidates include cystatin C, urinary N-acetyl-β-D-glucosaminidase, interleukin-18, kidney injury molecule-1, urinary neutrophil gelatinase-associated lipocalin and fatty acid-binding proteins, although further validation remains necessary [14].

## 2. Pathophysiology of Hepatorenal Syndrome (HRS)

HRS arises from several converging mechanisms in cirrhosis. Key drivers include circulatory dysfunction with splanchnic vasodilation and reduced effective arterial filling; a systemic inflammatory milieu often precipitated by bacterial infection and failure of renal autoregulation with tubular dysfunction; and maldistribution of intrarenal blood flow [15,16,17]. Additional contributors are hepatorenal reflex-mediated neurohumoral activation, relative adrenal insufficiency, bile acid-related renal and vascular effects and intra-abdominal hypertension, which further compromise renal perfusion [18,19].

Prerenal failure is acute kidney dysfunction due to renal hypoperfusion without intrinsic cellular injury [20]. It arises abruptly after physiological insults, classical hypovolemia from esophageal variceal bleeding that reduces effective circulating volume and renal perfusion or any event that acutely compromises renal oxygen delivery [21]. The course spans hours to days and features rising serum urea/creatinine with decreased urine output, manifesting as anuria or oliguria (<500 mL/day) [21]. In cirrhosis, susceptibility is amplified by hypovolemia, septic shock, major surgery and nephrotoxic exposures, with additional contributions from lactulose-induced diarrhea and poor oral intake [22]. The predominant precipitant in this setting remains diuretic-related hypovolemia during attempts to mobilize tense or large-volume ascites [23].

**Figure 1:**
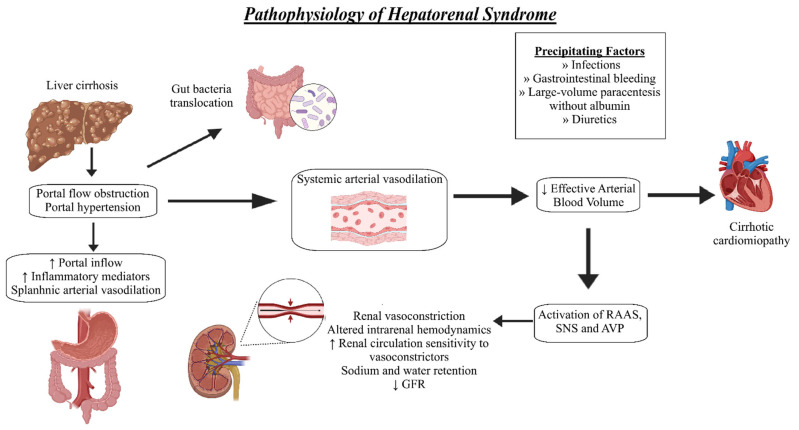
Pathophysiology of hepatorenal syndrome (HRS).

In patients with liver cirrhosis, structural remodeling and architectural distortion of the liver elevate intrahepatic vascular resistance, impeding portal venous inflow [24]. At the same time, increased shear stress within the splanchnic circulation induces the release of potent vasodilators, most notably nitric oxide, driving marked splanchnic vasodilation [25]. The conjunction of augmented splanchnic inflow from vasodilation with heightened intrahepatic resistance culminates in portal hypertension [26]. Because the diseased liver clears these vasodilators less efficiently, a proportion escapes into the systemic circulation through portosystemic shunts, producing systemic vasodilation [27,28]. Resultant splanchnic pooling effectively sequesters blood from the central circulation; coupled with the rise in systemic vascular capacitance due to arterial vasodilation, this generates arterial underfilling despite no true loss of intravascular volume [24]. The hemodynamic picture is one of systemic hypotension and renal hypoperfusion (Figure 1).

To counter the fall in mean arterial pressure, arterial baroreceptors activate endogenous vasoconstrictor pathways, including the nonosmotic vasopressin release, sympathetic nervous system (SNS) and the renin–angiotensin–aldosterone system (RAAS) [28,29]. Additional mediators, endothelin-1, thromboxane A2 and adenosine, also contribute to the pathogenesis of HRS-AKI [30]. The kidneys are particularly sensitive to SNS- and RAAS-mediated vasoconstriction, leading to intrarenal vasoconstriction and hypoperfusion, which is further compounded by impaired autoregulation [3]. This impairment reflects a rightward shift in the renal autoregulatory curve such that, for any given renal perfusion pressure, sympathetic overactivity yields lower renal blood flow [28]. The ensuing hypoperfusion reduces the glomerular filtration rate increases the risk of AKI [30,31,32]. A superimposed hemodynamic insult can then precipitate HRS-AKI. Prolonged SNS/RAAS activation with persistent renal hypoperfusion may provoke ischemia, microvascular endothelial injury and other irreversible cortical damage [30]. This framework describes a continuum: from HRS–acute kidney disease to HRS–AKI and, ultimately, HRS–chronic kidney disease, emphasizing the need for early detection and treatment of HRS–AKI.

## 3. Diagnostic Approach

### 3.1. Renal Biomarkers

Renal biomarkers used in cirrhosis are best conceptualized in three complementary classes: functional measures of filtration, indicators of tubular epithelial injury and markers of cell-cycle arrest, each illuminating a different facet of kidney dysfunction in this population [33]. Table 1 summarizes key renal biomarkers used in liver cirrhosis and AKI [12,14,32,33,34,35,36,37,38,39,40,41,42,43,44,45,46,47,48,49,50,51,52,53].

#### 3.1.1. Functional Biomarkers

Serum creatinine remains the frontline indicator for detecting declines in kidney function under current KDIGO and International Club of Ascites recommendations [12,54]. However, in cirrhosis, it systematically overestimates glomerular filtration because measured concentrations are artifactually low, driven by sarcopenia, increased proximal tubular secretion, bilirubin interference with the Jaffe assay and dilution from overhydration [12,34,55,56,57]. Enzymatic creatinine assays and rate-blanked Jaffe methods mitigate but do not eliminate bilirubin/chromogen interference, so results regarding cirrhosis require cautious interpretation [34,55,56,57]. For longitudinal decisions, the assay type (Jaffe vs. enzymatic) should be reported and kept constant when possible, and interpretation should prioritize creatinine kinetics (e.g., a rise ≥ 0.3 mg/dL within 48 h) rather than static thresholds in cirrhosis-related acute kidney injury [58].

When creatinine-based estimates are unreliable, particularly in cachectic patients or at moderate levels of kidney function, cystatin C-based equations provide superior fidelity because they are independent of muscle mass, less influenced by age and race and more informative within an estimated glomerular filtration rate of 60–90 mL/min/1.73 m^2^ [35,59,60]. Cystatin C is measured by standardized immunonephelometric or immunoturbidimetric assays, and a discrepancy in which eGFR based on cystatin C is substantially lower than eGFR based on creatinine often unmasks reduced filtration in sarcopenic cirrhosis and should prompt earlier escalation and consideration of transplant planning [33]. Nevertheless, neither biomarker is pathognomonic: both creatinine and cystatin C are modulated by nonrenal determinants. Therefore, findings should be judged against the complete clinical picture, not considered in isolation [35,59,60].

#### 3.1.2. Biomarkers for Tubular Injury

Beyond filtration, tubular injury biomarkers refine the differential diagnosis between functional hypoperfusion, HRS and structural acute tubular necrosis [19]. Neutrophil gelatinase-associated lipocalin (NGAL) rises rapidly after tubular epithelial injury, is typically highest in acute tubular necrosis (intermediate in HRS, lowest in prerenal states), carries independent prognostic information and appears relatively insensitive to volume status or diuretics, although urinary tract infection can artifactually elevate levels and must be excluded [36,61,62,63]. A systematic review and meta-analysis in cirrhosis found urine NGAL to robustly distinguish ATN from HRS/prerenal AKI and carry independent prognostic information for short-term outcomes, while another meta-analysis that added post-2016 cohorts confirmed urine NGAL as a leading discriminator of ATN in cirrhosis-associated AKI [64,65].

Interleukin-18 similarly tracks tubular damage and often follows the same gradient (acute tubular necrosis > HRS > prerenal), but its diagnostic specificity is tempered by extrarenal inflammatory drivers such as systemic inflammation, urinary tract infection, sepsis and ischemia–reperfusion; cautious clinical interpretation is therefore essential [36,37,38,39,66]. Urinary interleukin-18 reflects inflammasome-mediated tubular injury and, when interpreted together with neutrophil gelatinase-associated lipocalin, strengthens attribution to structural injury in cirrhosis-associated acute kidney injury [64]. Higher urinary interleukin-18 concentrations associate with worse short-term outcomes, supporting its use for early risk stratification in this population [64].

Trefoil factor-3 increases across AKI phenotypes (prerenal < HRS < acute tubular necrosis) but may be amplified by acute-on-chronic liver failure, limiting specificity [41,42]. Trefoil factor-3 indexes epithelial repair/remodeling and increases with tubular injury, supporting a structural phenotype when values are elevated [67]. Diagnostic utility improves when the biomarker is read alongside a direct tubular-injury signal such as neutrophil gelatinase-associated lipocalin, rather than in isolation [68,69,70].

Osteopontin varies with AKI subtype (lowest in prerenal, intermediate in HRS, highest in acute tubular necrosis), yet is modulated by hormonal/dietary factors and comorbidities [43,44,45,46]. Osteopontin reflects inflammatory–repair signaling in the tubulointerstitium and tends to be higher with structural tubular injury, while elevated levels in acute kidney injury also track worse survival and renal outcomes [44,71].

Monocyte chemoattractant protein-1 rises with AKI severity (stages 1–3) and typically follows the gradient acute tubular necrosis > HRS > prerenal, supporting etiologic differentiation in cirrhosis [47,48]. Urinary monocyte chemoattractant protein-1 indicates intrarenal chemokine activation, with higher levels aligning with structural injury and associating with adverse outcomes in decompensated cirrhosis [72].

Calbindin is predominantly elevated in acute tubular necrosis, rises less in hepatorenal or prerenal injury and shows no consistent increase in acute-on-chronic liver failure, features that may enhance utility in cirrhosis [41,49]. Urinary calbindin reflects distal-tubule involvement and rises early with tubular injury, showing higher values in structural than functional phenotypes and, when concordant with other markers, supports attribution toward acute tubular necrosis while complementing neutrophil gelatinase-associated lipocalin or kidney injury molecule-1 in multimarker panels [67].

Urinary glutathione-S-transferases (α/π) signal tubular epithelial damage and tend to peak in acute tubular necrosis, but their discriminatory accuracy is generally inferior to NGAL for distinguishing hepatorenal and prerenal states [50,51,52,53]. Segment-specific isoenzymes, α (proximal) and π (distal), help localize the site of tubular epithelial damage and tend to peak in structural injury rather than functional states [73]. Elevated urinary biomarker predicts more advanced AKI, reinforcing its role as a structural injury signal alongside higher-performing discriminators [53].

Toll-like receptor-4 expression in renal tubules and its urinary excretion increase with renal dysfunction and systemic inflammation in cirrhosis, and may become abnormal before creatinine rises [32,74]. Urinary Toll-like receptor-4 is increased in acute deterioration of cirrhosis with acute kidney injury compared with stable cirrhosis and controls, but because it rises with infection/inflammation, it should be interpreted alongside structural injury markers [75].

Urinary β2-microglobulin, reflecting tubular injury and immune activation, is usually highest in acute tubular necrosis and lower in prerenal or hepatorenal states, although isolated glomerular disease can also elevate it [41,42]. Urinary beta-2-microglobulin indicates proximal tubular reabsorptive failure and rises with structural tubular injury, with reliable interpretation requiring near-neutral urine pH during pre-analytical handling because the molecule degrades in acidic conditions [76,77].

Kidney injury molecule-1 (KIM-1), upregulated after ischemic/toxic proximal-tubule injury, supports the identification of structural damage (generally highest in acute tubular necrosis, modest in HRS and minimal in prerenal injury) [40,41]. Yet, on its own, it rarely achieves clean separation between structural and functional phenotypes [40,41]. Urinary KIM-1 increases with proximal tubular structural injury and is higher in acute tubular necrosis than in hepatorenal or prerenal states [78]. Combined with neutrophil gelatinase-associated lipocalin or interleukin-18, KIM-1 strengthens attribution to structural injury in cirrhosis-associated acute kidney injury [79].

Liver-type fatty-acid binding protein (L-FABP), a proximal-tubule protein induced by hypoxia and oxidative stress, typically mirrors these gradients (acute tubular necrosis > HRS > prerenal) and has been linked in exploratory work to subsequent HRS risk, adding a pathophysiologic lens to early risk stratification [36,40,61]. Urinary liver-type fatty-acid binding protein, measured by ELISA, reflects proximal-tubule hypoxic/oxidative stress and rises early across AKI phenotypes in cirrhosis, with higher values in acute tubular necrosis than in hepatorenal or prerenal states [80]. Higher urinary L-FABP levels are associated with worse short-term outcomes and the development of acute-on-chronic liver failure, providing prognostic information complementary to tubular-injury markers [80,81].

Finally, albuminuria, primarily a marker of glomerular or mixed (glomerular + tubular) injury, tends to be greatest in acute tubular necrosis and lower in HRS, aiding the structural–functional distinction at the bedside [40,41,42].

### 3.2. Prerenal vs. Postrenal AKI

Prerenal AKI accounts for ~46–66% of acute renal dysfunction in cirrhosis and is typically reversible with effective intravascular volume expansion, an approach that concurrently helps exclude HRS–AKI when renal indices promptly improve [28,82]. Susceptibility rises with diuretic therapy, gastrointestinal fluid depletion from laxatives used to prevent hepatic encephalopathy, and large-volume paracentesis without albumin replacement [28,82]. Systemic factors, especially heart failure with reduced forward output and the resulting upregulation of the renin–angiotensin–aldosterone axis and sympathetic tone, further compromise renal perfusion [54,82,83]. Medication review is essential: misdosed beta-blockers may aggravate cardiac depression and renal hypoperfusion via reflex neurohormonal activation, while sodium–glucose cotransporter-2 inhibitors can induce intravascular volume depletion and are linked to urinary tract infections (including urosepsis/pyelonephritis) [84,85,86]. Large cardiovascular outcome trials report similar AKI rates to placebo and dehydration from osmotic diuresis is a plausible mechanism when AKI occurs [84,85,86].

Volume expansion carries risks in advanced heart failure and severe renal impairment [85]. The International Club of Ascites advises a pragmatic two-day fluid challenge (no single definitive metric for volume status) and bedside ultrasonography of the inferior vena cava diameter and collapsibility index can refine triage [12,87]. In a pilot cohort with cirrhosis and AKI, 28% were fluid-depleted, 8% had intra-abdominal hypertension, 11% were fluid-expanded and only 36% were euvolemic; targeted therapy (albumin for depletion, loop diuretics for expansion, paracentesis for intra-abdominal hypertension) yielded renal improvement in 30% of overhydrated patients [12,19,88]. A biomarker check can also assist: normal urinary neutrophil gelatinase-associated lipocalin with a rising serum creatinine favors functional (prerenal) impairment rather than tubular injury, whereas fractional excretion indices perform poorly for separating prerenal AKI from HRS–AKI [36,62,82].

Obstructive AKI results from impaired urinary drainage, commonly due to retroperitoneal fibrosis, neurogenic bladder, prostatic hyperplasia or urinary tract malignancy, and may be heralded by urgency (notably with prostatic disease), polyuria, stones, tumors or gross hematuria [89,90]. Renal ultrasonography should be obtained in all AKI presentations to exclude obstruction, with pelvic/abdominal computed tomography or magnetic resonance imaging when needed to uncover alternative obstructing lesions (e.g., pelvic tumors, retroperitoneal fibrosis) [54]. No biomarker has adequate performance for diagnosing postrenal AKI, so diagnosis rests on history, examination and imaging [89,90].

### 3.3. Distinguishing HRS–AKI from ATN–AKI

Acute tubular necrosis–AKI typically fails to improve with plasma expansion and often necessitates renal replacement therapy, underscoring the importance of early categorization and timely treatment [12,54,91]. Panels of tubular-injury biomarkers: kidney injury molecule-1, interleukin-18, liver-type fatty acid-binding protein and neutrophil gelatinase-associated lipocalin show promise for distinguishing structural ATN-AKI from functional HRS–AKI, although evidence remains limited and validated cut-offs are needed [32,36,41,74,82]. Additional candidates (trefoil factor-3, glutathione-S-transferases, osteopontin, Toll-like receptor-4, and monocyte chemoattractant protein-1) come from small studies and require further validation in cirrhosis [32,36,41,74,82].

Higher fractional excretion of sodium and higher fractional excretion of urea support a diagnosis of acute tubular necrosis-related AKI (reflecting impaired tubular reabsorption), whereas low values favor preserved tubular function [92]. In severe cirrhosis, renal hypoperfusion and reduced glomerular filtration of sodium can lower the fractional excretion of sodium and although this measure generally performs better than the fractional excretion of urea, both are inferior in diagnostic accuracy to newer biomarkers, particularly neutrophil gelatinase-associated lipocalin [82,93,94]. In practice, a pattern of elevated biomarkers associated with acute tubular necrosis together with an increased fractional excretion of sodium supports acute tubular necrosis-related AKI over HRS-related AKI, but standardized thresholds and prospective validation remain priorities [41,82,94].

### 3.4. Artificial Intelligence in Cirrhosis-Related AKI and HRS

Artificial intelligence (AI) methods have begun to aid early AKI prediction, HRS phenotyping and short-term risk stratification in cirrhosis, but current evidence is preliminary and requires external validation. Most published studies are retrospective and frequently single-center, with scarce external/temporal validation and limited calibration reporting, which constrains bedside adoption [95]. Early studies used automated phenotyping on electronic records to identify HRS cases reproducibly, demonstrating the feasibility of informatics pipelines in this domain [96].

Unsupervised machine learning has been applied to large U.S. inpatient datasets to subtype HRS into clinically distinct clusters with different in-hospital mortality, supporting the biological and prognostic heterogeneity of HRS beyond a single “functional” label. In a national sample of 5564 primary HRS admissions (2003–2014), consensus clustering identified four phenotypes with graded outcomes, illustrating how data-driven subgroups may inform individualized management [97].

Supervised models trained on ICU cohorts (MIMIC-III/IV) can predict AKI onset or short-term mortality in cirrhosis using routinely captured labs and vitals, outperforming conventional scores in internal testing but needing robust temporal and external validation [98,99]. Domain-specific work has also targeted HRS risk prediction per se, including models built across MIMIC-IV and electronic Intensive Care Units, but these tools are not ready for bedside triage without prospective impact studies and calibration across centers [95,100]. Emerging reports describe HRS prediction pipelines with explainability components; however, the absence of pre-registered protocols and prospective clinical integration remains a key barrier [101].

Beyond prediction, AI in hepatology has expanded to broader prognostication and care optimization in cirrhosis, underscoring methodological lessons relevant to HRS (feature drift, interpretability and transportability) [102,103,104]. Contemporary reviews in hepatology and medical informatics emphasize heterogeneous modeling approaches, frequent lack of external validation and the need for transparent, explainable methods aligned with clinical workflows [105,106,107].

AI for HRS/AKI in cirrhosis is promising for earlier signal-detection of tubular injury risk, data-driven phenotyping to personalize vasoactive strategies and dynamic mortality risk monitoring [100,108,109]. Near-term priorities are multicenter prospective validation, harmonized feature sets and predefined clinical use-cases.

## 4. Management Algorithm

HRS is a pivotal complication in the natural history of decompensated advanced liver disease, conferring substantial mortality and a poor prognosis and affecting nearly one-half of patients hospitalized with cirrhosis [110]. Before instituting disease-specific vasoactive therapy, the AKI management algorithm mandates a systematic medication review with discontinuation of all potential nephrotoxins, including diuretics, non-selective beta-blockers and non-steroidal anti-inflammatory drugs, together with early identification and treatment of bacterial infections and prompt plasma volume expansion with albumin [12].

**Figure 2:**
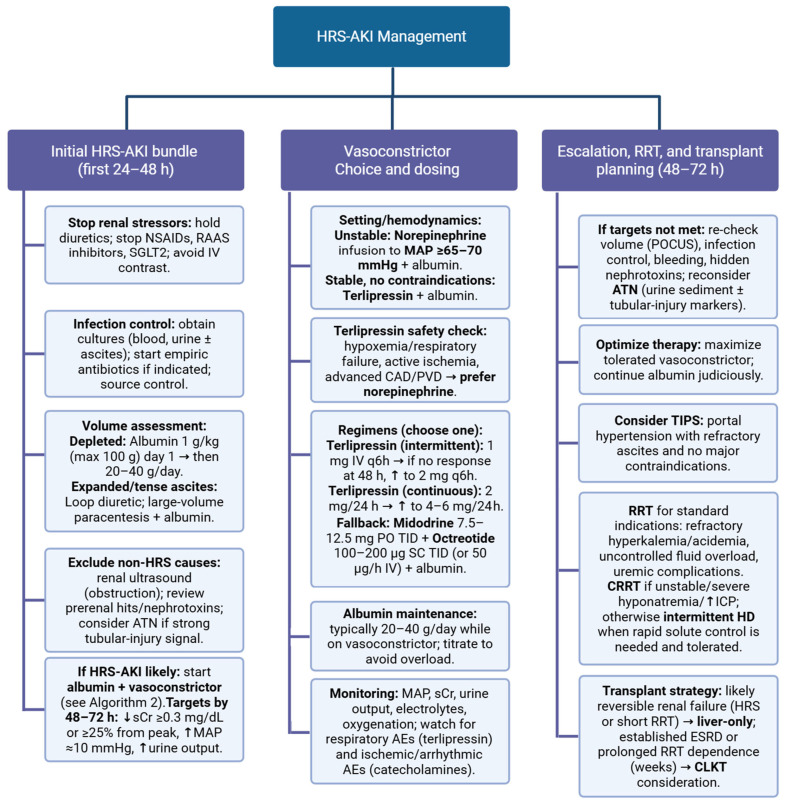
HRS-AKI Management. Abbreviations: HRS—Hepatorenal Syndrome; AKI—Acute Kidney Injury; ATN—Acute Tubular Necrosis; sCr—Serum Creatinine; MAP—Mean Arterial Pressure; RRT—Renal Replacement Therapy; CRRT—Continuous Renal Re-placement Therapy; HD—Hemodialysis; TIPS—Transjugular Intrahepatic Portosystemic Shunt; ESRD—End-Stage Renal Disease; CLKT—Combined Liver–Kidney Transplantation; NSAIDs—Nonsteroidal Anti-Inflammatory Drugs; RAAS—Renin–Angiotensin–Aldosterone System; SGLT2—Sodium–Glucose Cotransporter-2; IV—Intra-venous; PO—By mouth; SC—Subcutaneous; TID—Three times daily; q6h—Every 6 h; CAD—Coronary Artery Disease; PVD—Peripheral Vascular Disease; ICP—Intracranial Pressure; AE/AE—Adverse Event.

A broad therapeutic armamentarium is used to manage HRS (Figure 2). Strategies include resolution of alcoholic hepatitis, effective antiviral therapy in decompensated hepatitis B, recovery from acute hepatic failure and evaluation for liver transplantation [111,112]. When immediate improvement in hepatic function is unlikely, the kidney injury associated with HRS requires targeted medical management [113]. Withdrawal of antihypertensive agents, beta-blockers included, is recommended for all patients with HRS [58].

For individuals in the intensive care unit, initial therapy should pair norepinephrine with intravenous albumin [114]. Outside the intensive care setting, access to specific agents determines the preferred approach: where available, terlipressin plus albumin is advised as first-line therapy; if terlipressin cannot be used, a regimen combining midodrine, octreotide, and albumin is an accepted alternative [114]. In selected nonresponders who meet eligibility criteria, placement of a transjugular intrahepatic portosystemic shunt (TIPS) has shown benefit. For patients who fail to respond, have severe renal impairment and are either candidates for liver transplantation or have a reversible hepatic insult with a reasonable chance of survival, dialysis becomes a consideration, as a temporizing strategy toward liver transplantation or possible hepatic recovery [115].

The therapeutic cornerstone in HRS-AKI is vasoconstrictor therapy combined with albumin [116]. In the phase-3 CONFIRM trial, terlipressin plus albumin achieved a higher verified reversal rate than placebo plus albumin, without a 90-day survival advantage [117]. Head-to-head evidence syntheses indicate no statistically significant difference between terlipressin and norepinephrine for HRS reversal or short-term mortality when both are co-administered with albumin [118,119].

Accordingly, either agent is acceptable: norepinephrine is practical first-line in the ICU (titrated to a target MAP), whereas terlipressin is reasonable on the ward when respiratory risk is low and monitoring is available [116]. A key limitation of terlipressin is a higher rate of respiratory adverse events noted in randomized data [120]. Where preferred options are not feasible, midodrine plus octreotide plus albumin can be used on the ward, but randomized evidence shows inferior reversal compared with terlipressin-based therapy [121].

Recent clinical reviews similarly judge the midodrine–octreotide combination less effective than terlipressin (and inferior to catecholamines) for reversal [122]. In selected non-responders with portal-hypertension physiology and refractory ascites, TIPS may improve renal indices by reducing splanchnic congestion, though evidence is largely observational and careful candidate selection is essential [123].

Renal replacement therapy (RRT) addresses life-threatening complications and serves as a bridge rather than a disease-modifying therapy; modality choice hinges on hemodynamic stability [115]. RRT, most often intermittent hemodialysis, is frequently required as orthotopic liver transplantation (OLT) candidates become more overtly decompensated [124]. Outcomes are poor once dialysis is needed before transplant: only 35% of patients on pre-OLT RRT survive to transplantation or discharge, whereas 65% die while waiting; among those who do undergo OLT, 1-year mortality is markedly higher when RRT was initiated preoperatively (30% vs. 9.7% without pre-OLT RRT) [125].

Indications in end-stage liver disease are the usual ones: refractory acidemia, serious electrolyte abnormalities, uncontrolled fluid overload or uremic symptoms despite medical therapy [126,127]. Cirrhosis introduces several diagnostic and management pitfalls. First, chronic respiratory alkalosis with a superimposed metabolic compensation may mimic primary acidemia unless arterial blood gas analysis is performed to define the dominant acid–base process [128]. Second, uremic encephalopathy can be clinically indistinguishable from hepatic encephalopathy, necessitating a structured differential diagnosis that considers renal failure-related neurotoxicity alongside liver-related causes [129]. Third, patients with cirrhosis are hemodynamically fragile and frequently develop intradialytic hypotension, a feature that often favors continuous kidney replacement therapy over intermittent hemodialysis when instability is present [124,130].

Continuous kidney replacement therapies are preferred in the setting of profound hypotension, severe hyponatremia requiring controlled correction or elevated intracranial pressure with cerebral edema because solute and fluid shifts can be distributed over twenty-four hours rather than compressed into a brief session [91,130]. Conversely, when life-threatening biochemical derangements demand rapid clearance, such as classically severe hyperkalemia or marked acidemia, intermittent hemodialysis is favored for its superior rate of solute removal [131,132]. Albumin-based or hybrid extracorporeal systems and fractionated plasma separation and adsorption may serve as bridges to OLT, but their routine role remains unproven and requires stronger evidence [133].

Against this backdrop, adoption of the end-stage liver disease allocation model in 2002 coincided with a tripling of combined liver–kidney transplantation (CLKT), from 134 procedures in 2001 to 399 in 2006, underscoring the central influence of creatinine in organ prioritization [134]. CLKT confers superior 1-year survival compared with liver-only transplantation when true end-stage renal disease (ESRD) is present, but offers no survival advantage in candidates without ESRD, raising concern that CLKT may be offered to patients whose renal failure is potentially reversible (notably HRS or acute tubular necrosis) and might recover after OLT [135]. While renal biopsy remains the definitive method for determining chronicity, it is often impractical in cirrhosis with coagulopathy; intraoperative renal biopsy has been proposed to guide CLKT necessity, yet no consensus or criteria exist for patient selection [135].

In practice, noninvasive renal ultrasonography (kidney size, cortical echogenicity) helps gauge reversibility, and a pragmatic signal is dialysis dependence for more than several weeks before OLT, which predicts a low likelihood of post-transplant renal recovery and prompts serious consideration of CLKT. In 2007, an expert panel convened to refine indications, create a registry and recommend standardized listing criteria, aiming to balance timely transplantation against the risk of unnecessary kidney allocation [134,135,136]. Table 2 summarizes recent evidence on HRS and AKI in cirrhosis.

## 5. Conclusions

HRS exemplifies a predominantly functional, potentially reversible kidney failure that emerges from a tight hemodynamic–inflammatory coupling in cirrhosis. Splanchnic vasodilation with effective arterial underfilling and neurohumoral upregulation remains the core paradigm, while systemic inflammation and microcirculatory dysfunction explain the clinical variability and frequent nonresponse to purely hemodynamic interventions. Recent definitional updates have shifted emphasis from static creatinine thresholds to dynamic trajectories, enabling earlier recognition of HRS–AKI. In practice, this demands protocolized surveillance of creatinine kinetics in hospitalized patients with decompensated cirrhosis and rapid exclusion of postrenal and overt prerenal causes.

Differentiation of HRS–AKI from acute tubular necrosis is the pivotal diagnostic fork because it redirects therapy. A tiered approach that couples baseline tests (serum creatinine, cystatin C), structured volume assessment (albumin challenge, bedside ultrasound) and a limited biomarker panel offers clinically actionable discrimination without overcomplexity. Fractional excretion indices add context but should not supersede biomarker and clinical data. Management should be early, bundled and goal-directed: stop nephrotoxins, treat infection and initiate albumin plus a vasoconstrictor (terlipressin where available; norepinephrine in the ICU; midodrine/octreotide when other options are not feasible). For nonresponders, timely consideration of TIPS and individualized dialysis modality selection based on hemodynamic stability are essential bridges to either recovery or transplantation.

Transplant strategy must align organ stewardship with patient outcomes. Combined liver–kidney transplantation improves survival when end-stage renal disease is established, but in potentially reversible renal failure (HRS or short-duration dialysis), isolated liver transplantation should remain the default pending objective evidence of chronicity (imaging, trajectory and—where safe—biopsy). The research agenda should prioritize validated cut-offs for tubular-injury biomarkers, prospective algorithms that integrate biomarkers with ultrasound-based volume profiling and trials testing standardized, time-to-treatment pathways. Closing these gaps is likely to reduce diagnostic delay, optimize resource use, and measurably improve survival in this high-risk population.

## Figures and Tables

**Table 1:** Renal biomarkers in hepatorenal syndrome.

Class	Biomarker	Biological Source	Typical Pattern Across AKI Phenotypes	Clinical Utility	Main Confounders/Caveats
Functional	**Serum creatinine (sCr)**	Muscle creatine/creatine phosphate → creatinine; filtered and secreted in proximal tubule	Falls with true ↓GFR, but may be falsely low in cirrhosis	Cornerstone for AKI dx; widely used for eGFR; may overestimate kidney function in cirrhosis	Sarcopenia, ↑tubular secretion, bilirubin interference (Jaffe), overhydration; enzymatic assays/rate-blanking mitigate but do not eliminate bias
	**Cystatin C**	Constant production by all nucleated cells; freely filtered, no tubular secretion	Tracks ↓GFR (often more reliably than sCr at eGFR 60–90)	Better when muscle mass is low; generally more accurate in advanced liver disease	Still affected by nonrenal factors (e.g., thyroid, inflammation, steroids)
Tubular injury	**NGAL**	Loop of Henle and collecting duct (also neutrophils); released with tubular injury	Highest in ATN, intermediate in HRS, lowest in prerenal	Early rise; prognostic value; relatively insensitive to volume/diuretics	Urinary tract infection can raise levels
	**IL-18**	Interstitial macrophages; proximal tubule and collecting duct; inflammasome-dependent release	ATN > HRS > prerenal	Marker of tubular injury; aids ATN vs. HRS/prerenal	Sepsis, systemic inflammation, UTI, ischemia–reperfusion can elevate
	**L-FABP**	Proximal tubule; induced by hypoxia/oxidative stress	ATN > HRS > prerenal	Reflects tubular stress/injury; exploratory links to later HRS risk	Similar confounders as other tubular markers
	**KIM-1**	Proximal tubular epithelium (upregulated after ischemic/toxic injury)	ATN > HRS > prerenal	Indicates structural tubular damage; helps ATN vs. functional	Limited power alone to cleanly separate HRS from ATN
	**TFF-3**	Collecting duct epithelium; epithelial repair peptide	ATN > HRS > prerenal	Supports phenotyping of AKI type	May be amplified in acute-on-chronic liver failure
	**Calbindin**	Distal nephron; Ca^2+^-binding protein	Highest in ATN; low in HRS/prerenal	Useful adjunct in cirrhosis; not increased by ACLF	Limited evidence base
	**GST-α/π**	Proximal (α) and distal (π) tubular enzymes	Peak in ATN	Detect tubular epithelial injury; adjunctive	Generally less accurate than NGAL for HRS vs. prerenal
	**Osteopontin (OPN)**	Loop of Henle and collecting ducts; immune cells	ATN > HRS > prerenal	Diagnostic/prognostic potential; reflects inflammation/repair	Modulated by hormones, diet, comorbidities
	**MCP-1**	Chemokine produced during renal inflammation	Typically ATN > HRS > prerenal; rises with AKI stage	Aids etiologic differentiation in cirrhosis	Inflammation-driven; interpret with context
	**TLR4 (urinary)**	Upregulated in renal tubules; shed into urine	Elevates with tubular injury/inflammation; may precede sCr rise	Early signal of injury in cirrhosis + systemic inflammation	Also rises from systemic inflammatory states
	**β2-microglobulin (urinary)**	Filtered at glomerulus; reabsorbed in proximal tubule	Usually highest in ATN; lower in HRS/prerenal	Indicates proximal tubular dysfunction	Can rise in isolated glomerular disease
Glomerular/mixed	**Urinary albumin**	Glomerular leakage; also rises with mixed glomerular + tubular injury	Highest in ATN (esp. mixed injury); lower in HRS	Helps distinguish structural (ATN) from functional (HRS/prerenal)	Proteinuria from primary glomerular disease

Abbreviations: AKI—Acute Kidney Injury; HRS—Hepatorenal Syndrome; ATN—Acute Tubular Necrosis; sCr—Serum Creatinine; eGFR—Estimated Glomerular Filtration Rate; NGAL—Neutrophil Gelatinase-Associated Lipocalin; IL-18—Interleukin-18; L-FABP—Liver-type Fatty Acid-Binding Protein; KIM-1—Kidney Injury Molecule-1; TFF-3—Trefoil Factor-3; GST-α/GST-π—Glutathione-S-Transferase alpha/pi; OPN—Osteopontin; MCP-1—Monocyte Chemoattractant Protein-1; TLR4—Toll-Like Receptor 4; ACLF—Acute-on-Chronic Liver Failure.

**Table 2:** Hepatorenal syndrome and acute kidney injury in cirrhosis—comparative evidence.

Citation	Study Design	Population	Key Outcomes	Clinical Takeaway
Tangpanithandee et al., 2023 [97]	Unsupervised clustering of national inpatient data	5564 HRS admissions	Four HRS phenotypes with graded in-hospital mortality; highest risk in the multi-organ failure/ventilation/RRT cluster.	Risk-stratify by phenotype; escalate monitoring and treatment in high-risk groups.
Yoo et al., 2021 [137]	Multicenter prospective cohort	262 cirrhosis + AKI (HRS-AKI n = 73)	Urinary NAG increases with AKI severity and predicts 3-month outcomes; it does not predict terlipressin response.	Use NAG for prognosis, not to select vasoconstrictor therapy.
Abboud et al., 2024 [138]	Single-center retrospective cohort	140 cirrhosis + AKI	AKI resolution associated with higher admission albumin and non-MASLD etiology; higher creatinine predicted non-resolution; resolution improved survival.	Correct reversible factors early; aim to achieve AKI resolution during index admission.
Wan et al., 2024 [118]	Systematic review and meta-analysis of RCTs	15 trials; N = 1236	Terlipressin+albumin improves HRS reversal vs. placebo; broadly similar efficacy to norepinephrine; no clear survival benefit; more adverse events with terlipressin.	Terlipressin or norepinephrine are both acceptable; monitor agent-specific AEs.
Olson et al., 2024 [119]	Meta-analysis of head-to-head RCTs	7 trials; N = 376	No statistically significant difference between terlipressin and norepinephrine; small numerical edge for terlipressin in reversal and 1-month survival.	Choose based on availability, care setting, and patient risk profile.
Farooq et al., 2024 [139]	Nationwide readmissions trend analysis	169,522 HRS admissions (2010–2018)	Rising HRS admissions and 30-day readmissions; infections and liver failure are leading causes.	Emphasize infection prevention and structured early post-discharge follow-up.
Yoshimura et al., 2024 [140]	Case report	1 patient (alcohol-related cirrhosis + IgAN)	Marked, sustained proteinuria reduction with dapagliflozin alongside abstinence.	Hypothesis-generating only; not practice-changing without trials.
Scarlata et al., 2024 [141]	Bicentric retrospective study	236 cirrhotics	MELD best predicted HRS; MELD-Na improved specificity; other indices had modest performance.	Use MELD/MELD-Na for routine HRS risk assessment.
Wong et al., 2021 [117]	Phase 3 RCT, double-blind	300 HRS-1	Verified HRS reversal: 32% terlipressin vs. 17% placebo; no 90-day survival benefit; more respiratory adverse events with terlipressin.	Screen for respiratory risk before terlipressin; reversal benefit without proven survival gain.
Park et al., 2015 [142]	Diagnostic DWI-MRI (3T) study	64 cirrhotics	Lower cortical/medullary ADC with impaired renal function; ADC correlates with eGFR/sCr; fair discrimination.	Consider DWI-MRI as a supportive tool where available; not standalone.
Sigal et al., 2023 [143]	Pooled subgroup from three phase-3 RCTs	~205 alcoholic hepatitis + HRS	Higher HRS reversal with terlipressin (~38% vs. ~13%); signal of greater benefit when Maddrey DF < 32.	Factor disease context (alcoholic hepatitis, DF) into expected benefit.

Abbreviations: HRS—Hepatorenal Syndrome; RRT—Renal Replacement Therapy; AKI—Acute Kidney Injury; NAG—N-Acetyl-β-D-Glucosaminidase; MASLD—Metabolic Dysfunction-Associated Steatotic Liver Disease; sCr—Serum Creatinine; RCT—Randomized Controlled Trial; AE–Adverse Event; SGLT2—Sodium–Glucose Cotransporter-2; IgAN—IgA Nephropathy; MELD—Model for End-Stage Liver Disease; MELD-Na—MELD with Serum Sodium; HRS-1—Type-1 Hepatorenal Syndrome; DWI—Diffusion-Weighted Imaging; MRI—Magnetic Resonance Imaging; ADC—Apparent Diffusion Coefficient; eGFR—Estimated Glomerular Filtration Rate; DF—Maddrey Discriminant Function.

## Data Availability

No new data were created or analyzed in this study. Data sharing is not applicable to this article.
